# Meta-analysis of magnetic resonance imaging accuracy for diagnosis of oral cancer

**DOI:** 10.1371/journal.pone.0177462

**Published:** 2017-05-24

**Authors:** Marcelo Aldrighi Moreira, Luiza Silveira Lessa, Francieli Regina Bortoli, Abigail Lopes, Eduardo Picolo Xavier, Renan Antonio Ceretta, Fernanda Guglielmi Faustini Sônego, Cristiane Damiani Tomasi, Patricia Duarte Simões Pires, Luciane Bisognin Ceretta, Ingrid Dalira Schweigert Perry, Priscyla Waleska Simões

**Affiliations:** 1 Graduate Program in Public Health (PPGSCol), Unidade Acadêmica de Ciências da Saúde (UNASAU), Universidade do Extremo Sul Catarinense (UNESC), Criciúma, SC, Brazil; 2 Laboratory of Information and Communications Technology in Health (TISaude), Universidade do Extremo Sul Catarinense (UNESC), Criciúma, SC, Brazil; 3 Dentistry Course, Unidade Acadêmica de Ciências da Saúde (UNASAU), Universidade do Extremo Sul Catarinense (UNESC), Criciúma, SC, Brazil; 4 Universidade Federal de Santa Catarina (UFSC), Florianópolis, SC, Brazil; 5 Engineering, Modeling and Applied Social Sciences Center (CECS), Universidade Federal do ABC (UFABC), São Bernardo do Campo, SP, Brazil; Waseda University, JAPAN

## Abstract

**Objective:**

To establish the diagnostic accuracy of magnetic resonance imaging (MRI) as an auxiliary means for the diagnosis of oral cancer through a systematic review and meta-analysis.

**Methods:**

An exhaustive search of publications from 1986 to 2016 was performed of Medline, Embase and Cochrane (and related databases), including grey literature. Primary diagnostic accuracy studies that assessed oral cancer (target condition) using MRI (index test) were included. Diagnostic threshold, sensitivity and meta-regression analyses were performed. A meta-analysis was performed using Meta-DiSc® v. 1.4 software.

**Results:**

A total of 24 primary studies were assessed, comprising 1,403 oral cancer lesions. Nine studies used diffusion-weighted MRI, with a diagnostic odds ratio (DOR) of 30.7 (95% confidence interval [CI]: 12.7–74.3) and area under the curve (AUC) of 0.917 (95% CI: 0.915–0.918); seven studies used dynamic contrast-enhanced MRI, with a DOR of 48.1 (95%CI: 22.4–103.2) and AUC of 0.936 (95% CI: 0.934–0.937); and 13 studies used traditional MRI, with a DOR of 23.9 (95%CI: 13.2–43.3) and AUC of 0.894 (95% CI: 0.894–0.895). Meta-regression analysis indicated that the magnetic field strength may have influenced the heterogeneity of the results obtained (p = 0.0233) using traditional MRI. Sensitivity analysis revealed a discrete reduction of inconsistency in some subgroups.

**Conclusion:**

The three types of MRI assessed exhibited satisfactory accuracy compared to biopsy. Considering the relevance of early treatment and screening and that better health care results in improved survival rates and quality of life for oral cancer patients, we suggest the use of MRI as a part of the pre-treatment and monitoring protocol at public health services.

## Introduction

Oral cancer is currently considered to be a public health problem worldwide. Although it represents little more than 2% of the global incidence of cancer, its lethality of 50% is a cause of much concern [1]; moreover, this type of cancer is the sixth most common globally [2]. Approximately 650,000 new cases occur every year, and approximately 40% of head and neck tumours are squamous cell carcinomas [3].

The high mortality rate of oral cancer has many causes and is likely associated with diagnostic delay. Oral lesions are easy to access and should be diagnosed early for treatment to be efficacious. Nevertheless, patients are often diagnosed in an advanced stage of disease. Diagnosis is late in most cases because patients do not seek treatment or do not have easy access to professionals who can establish the diagnosis [4].

Oral cancer has a subtle and asymptomatic onset, contributing to the diagnostic delay; for this reason, special attention is required from clinical practitioners, especially when risk factors, such as smoking, alcohol consumption and sun exposure, are present [5]. The prevention of oral cancer is intimately associated with early diagnosis and behavioural changes, such as quitting smoking and drinking [6].

Within this context, health services must simultaneously detect oral cancer in patients and maintain continued health education programmes that target risk factors. In addition, specific times and/or situations must be chosen to increase public opinion [7]. For example, dentists can play a significant role in the early detection of malignant and premalignant conditions and should assess all patients at high risk for disease [7, 8].

The current first line of investigation of oral abnormalities is visual inspection, which is a subjective method. Biopsy is the most widely accepted technique for accurate identification of lesions in the oral mucosa and is considered the gold standard for the detection of oral cancer [9].

Considering the aforementioned facts and the difficulties for early detection of oral cancer, the search for evidence and assessment of new rapid, precise and less invasive diagnostic methods is highly relevant [10]. Within this context, the use of imaging methods for the investigation of malignant head and neck tumours has increased substantially in parallel with the development of the modern, so-called last-generation methods [11].

Magnetic resonance imaging (MRI) is the technique that affords the highest-quality images of soft tissues without employing ionising radiation and without known biological risks [12]. This technique provides information on the extent of lesions, probable infiltration of major vessels and lymph node involvement, thus contributing to the determination of treatment and prognosis [13]. Some authors have noted the increasing use of diagnostic imaging techniques, such as MRI, among patients undergoing treatment for oral cancer [14–16].

One meta-analysis addressing this subject compared the accuracy of computed tomography and MRI for the diagnosis of cervical lymph node metastasis of head and neck cancer [17]. Another meta-analysis assessed the accuracy of MRI for the specific diagnosis of mandibular involvement of head and neck cancers [18]. However, none of these studies assessed the accuracy of MRI for the specific diagnosis of oral neoplasms.

Based on the aforementioned considerations and the lack of systematic reviews and meta-analyses assessing the accuracy of MRI for specific diagnosis of oral cancer, the present study sought to provide evidence on this subject. Oral cancer impacts the morbidity, mortality and quality of life of users of public health services, and early diagnosis can lead to a better prognosis. Moreover, methods such as MRI (i.e., non-invasive, easy to perform and reportedly efficacious for the diagnosis and precise delimitation of oral cancer lesions) are available at public health services. For these reasons, an assessment of the accuracy of these techniques for the diagnosis of oral cancer via a systematic review and meta-analysis will provide important evidence of their diagnostic validity. These data may serve as the foundation of proposals to include such methods in clinical practice, ultimately having a direct impact on the oral health of patients.

## Materials and methods

A systematic review of diagnostic accuracy studies was performed. An exhaustive search strategy of studies published from 1986 to 2016 was performed in the databases Medical Literature Analysis and Retrieval System Online (Medline) via PubMed **(S1 Fig),** Cancer Literature (CANCERLIT), Latin American and Caribbean Health Sciences Literature (LILACS), Excerpta Medical Database (Embase) and related databases, including so-called “grey” literature”, using items for Systematic Reviews and Meta-analysis (PRISMA) guidelines **(S1 File)**.

Search terms were selected from Medical Subject Headings (MeSH) and the Embase Emtree thesaurus and included “Oral Cancer” and synonyms associated to the index diagnostic test, which was designated “Magnetic Resonance” (and synonyms).

The symbol “*” was used to retrieve all variations of the search terms suffixes. The aforementioned terms were combined using the Boolean operators “AND”, “OR” and “NOT”.

The search was restricted to studies on humans; no limitation was established as to the language of publications. The references mentioned in all retrieved primary studies were analysed. In addition, authors of publications with incomplete data were directly contacted.

The titles and abstracts of retrieved studies were independently analysed by two investigators. Articles in English were assessed by two reviewers, and articles in other languages by a third independent reviewer; translations were performed when necessary. Instances of disagreement as to inclusion or exclusion of articles were solved by consensus; when consensus could not be attained, disagreement was solved by a fourth reviewer.

Primary diagnostic accuracy studies relating to diagnosis of oral cancer (target condition) by means of MRI (index test) were included. Studies reporting on oral cancer in adult patients were considered; oral cancer was defined as neoplasms affecting the lips and oral cavity (oral mucosa, gums, hard palate, tongue, salivary glands and oral floor). The MRI results (positive or negative) were considered as index tests.

Studies with oral cancer patients assessed based on the gold standard test (biopsy) and studies that included patients with histopathological diagnosis of oral cancer and without previous treatment were considered in the present review. Only studies in which the magnetic field strength (T) varied from 0.5 T to 3 T were considered because this is the most effective range for the identification of malignant tumours.

Studies with data that did not allow for the construction of a 2 x 2 contingency table were excluded, as were studies with patients who exhibited contraindications to MRI, including pacemakers, aneurysm clips, metallic fragments in the eyes, cochlear implants, ocular implants, internal drug infusion pumps, certain prosthetic heart valves, steel-containing bullets, bone growth stimulators and neurostimulators [6].

Studies conducted with patients exhibiting contraindications to the use of gadolinium contrast media were excluded. These conditions included the following: kidney failure, haemolytic anaemia, sickle-cell anaemia, pregnancy, breastfeeding, respiratory disorders, asthma and a history of allergy to the contrast media.

Next, the following data were extracted from the included studies: year of publication, country and continent where the study was conducted, type of oral cancer, study design and demographic data (e.g., age and gender) in addition to number of oral cancer patients and number of diagnostic hits on MRI.

These data were independently extracted by two reviewers. The data from articles published in languages other than English were independently extracted by a third reviewer; translations were performed when necessary. Instances of disagreement as to the extracted data were solved first by consensus or by another reviewer when consensus could not be reached.

The methodological quality of studies was assessed based on the criteria included in the Quality Assessment of Diagnostic Accuracy Studies 2 (QUADAS-2) tool [19, 20], which was designed to evaluate studies based on four key domains (patient selection; index test; reference standard; and flow and timing).

A 2 x 2 contingency table was constructed for each selected study; the results corresponding to the gold standard and MRI were entered as positive or negative. Sensitivity, specificity and likelihood ratio were calculated; the diagnostic odds ratio (DOR) was used as measure of diagnostic accuracy. A DOR value of 1 indicates a test without discriminatory power; the higher the DOR value, the greater the degree of relevance of the assessed diagnostic test [21].

For the studies in which one single cell in the 2 x 2 contingency table had a value of 0, 0.5 was added to all of the cells to enable calculation. However, studies in which 0 occurred in more than two cells were excluded from meta-analysis.

Meta-analysis was performed using software Meta-DiSc® version 1.4 (developed by Unit of Clinical Biostatistics, Ramón y Cajal Hospital, Madrid, Spain)[22]. Bivariate analysis was used to perform combined estimations of sensitivity, specificity and likelihood ratio with 95% confidence interval (CI) to estimate the summary results presented in the meta-analysis [23–25]. Summary measures were obtained using the DerSimonian and Laird’s random effects model (which considers the heterogeneity inherent to this type of systematic review) [26]. Heterogeneity was calculated using the chi-squared (*X*^2^) and Cochran’s Q test, inconsistency was assessed using *I*^2^, and *τ*^2^ was used to estimate variation between studies [23, 27].

Because of the heterogeneity that was found, sensitivity analysis was performed to identify associated cofactors (e.g., continent where the study was performed and the tumour primary site). Cofactors that were potentially associated with heterogeneity were also subjected to meta-regression [28].

To further investigate heterogeneity, diagnostic threshold analysis was performed based on the correlation (Spearman’s) between the logit of sensitivity and the logit of [1 –specificity] [22]. When a threshold effect occurs, the sensitivity and specificity of the investigated study exhibit negative correlation (or a positive correlation between sensitivity and [1 –specificity]. Therefore, a strong positive correlation between sensitivity and [1 –specificity] suggests the presence of a threshold effect [22].

When heterogeneity was observed, a summary receiver operating characteristic (SROC) curve was plotted. This method was appropriate given that the global sensitivity and specificity values were overestimated. In such cases, in addition to analysis of the ROC panel points, analysis of the SROC curve is also recommended [29, 30].

As is known, the various types of MRI techniques exhibit differences in threshold; therefore, to expand the information provided the study, the analysis was divided by MRI type (i.e., diffusion-weighted MRI [DW MRI], dynamic contrast-enhanced [DCE MRI] and traditional MRI).

The present study is registered at the PROSPERO database under number CRD42016043868.

## Results and discussion

**Fig 1:**
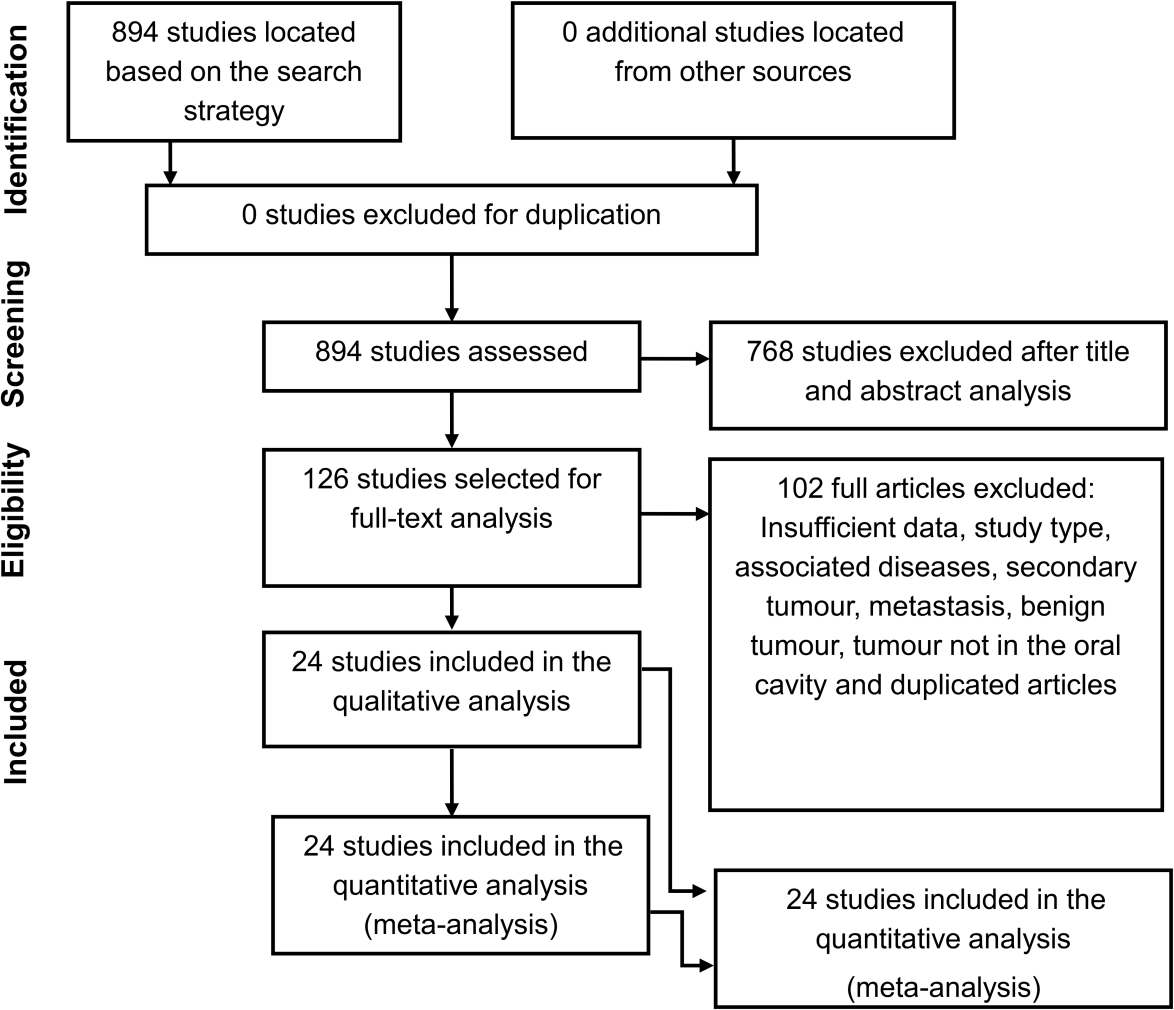
Study flow diagram.

A total of 24 studies on the use of MRI as a diagnostic test for oral cancer were included in the systematic review, comprising 1,404 oral cancer lesions [31–54]. The study selection process is depicted in **Fig 1**. Search in databases retrieved 894 abstracts. Following assessment of titles and abstracts, the full text of 126 studies was analysed, and 24 were selected for meta-analysis.

Nine studies performed DW MRI [32, 33, 36, 37, 41, 44, 49, 50, 52], seven used DCE MRI [31, 32, 39, 42, 43, 49, 53], and 13 applied traditional MR [33–35, 38, 40, 42, 45–48, 51, 52, 54]. It should be noted that some studies used more than one MRI type [32, 33, 42, 49, 52].

A full description of the studies, the standards and the tests used is presented in Table 1.

**Table 1:** Characteristics of included studies–MRIs.

Author/year	Country	Continent	Cancer cases	Lesion number	Not ill	Tumour site	Tumour type	Tesla	Design	Test site	Gender	Mean age	Total number of individuals	Prevalence
Aghaghazviniet al. 2015 (DCE MRI)[31]	Iran	Asia	10	46	36	Major salivary glands	Carcinoma and Adenocarcinoma	3.0 T	Retrospective	University	22 (Male) / 24 (Fem)	Not indicated	46	21.7%
Ai et al. 2013 (DCE and DW MRI)[32]	China	Asia	33	46	13	Tongue	Carcinoma	1.5 T	Retrospective	Hospital	23 (Male) / 10 (Fem)	35	33	71.7%
Alibek et al. 2007 (DW and traditional MRI)[33]	Germany	Europe	13	112	99	Parotid gland	Carcinoma	1.5 T	Retrospective	Not indicated	55 (Male) / 57 (Fem)	54	112	11.6%
Bartels et al. 2000 (traditional MRI)[34]	USA	America	17	35	18	Parotid gland	Carcinoma	Not indicated	Retrospective	Hospital	Not indicated	Not indicated	Not indicated	44.8%
Christe et al. 2011 (traditional MRI)[35]	USA	America	27	84	57	Parotid gland	Carcinoma	1.5 T	Retrospective	Hospital	43 (Male) / 41 (Fem)	56.0	84	32.1%
Eida et al. 2007 (DW MRI)[36]	Japan	Asia	9	31	22	Salivary glands	Carcinoma and Adenocarcinoma	1.5 T	Retrospective	Not indicated	13 (Male) / 18 (Fem)	63	31	29.0%
Fassnacht et al. 2013 (traditional MRI)[38]	Belgium	Europe	20	133	113	Parotid gland	Carcinoma	1.5 T/3 T	Retrospective	University	Not indicated	Not indicated	Not indicated	15.0%
Hisatomi et al. 2007 (DCE MRI)[39]	Japan	Asia	12	46	35	Salivary glands	Carcinoma	1.5 T	Retrospective	Hospital	15 (Male) / 21 (Fem)	56.8	36	26.0%
Inci et al. 2010 (DW MRI)[37]	Turkey	Asia	8	25	17	Parotid gland	Carcinoma	1.5 T	Retrospective	Not indicated	13 (Male) / 9 (Fem)	44	22	32.0%
Inohara et al. 2008 (traditional MRI)[40]	Japan	Asia	21	81	60	Parotid gland	Carcinoma	1.0T	Retrospective	Hospital	Not indicated	Not indicated	Not indicated	22.5%
Kato et al. 2015 (DW MRI)[41]	Japan	Asia	9	31	22	Parotid gland	Carcinoma	3 T	Retrospective	Hospital	17 (Male) / 14 (Fem)	63	31	29.0%
Lam et al. 2015 (DCE and traditional MRI)[42]	Japan	Asia	24	98	74	Salivary glands	Carcinoma and Adenocarcinoma	1.5 T	Retrospective	Hospital	36 (Male) / 62 (Fem)	53	98	24.4%
Matsuzaki et al. 2012 (DCE MRI)[43]	Japan	Asia	15	32	17	Minor salivary glands	Carcinoma	1.5 T	Retrospective	Not indicated	16 (Male) / 16 (Fem)	57.8	32	46.8%
Motoori et al. 2005 (DW MRI)[44]	Japan	Asia	5	33	28	Parotid gland	Carcinoma	1.5 T	Retrospective	Not Informed	26 (Male) / 7 (Fem)	60	33	15.1%
Paris et al. 2005 (traditional MRI)[45]	France	Europe	15	86	71	Parotid gland	Carcinoma	1.5 T	Retrospective	Hospital	83 (Male) / 98 (Fem)	55.2	181	17.4%
Prades et al. 2007 (traditional MRI)[46]	France	Europe	13	69	56	Parotid gland	Carcinoma	1.0T/1.5 T	Retrospective	Not indicated	29 (Male) / 39 (Fem)	55.1	68	19.1%
Rudack et al. 2007 (traditional MRI)[47]	Germany	Europe	30	109	79	Salivary glands	Not indicated	1.0T/1.5 T	Retrospective	Not indicated	61 (Male) / 48 (Fem)	55.4	109	27.5%
Sakamoto et al. 2014 (traditional MRI)[48]	Japan	Asia	34	100	66	Parotid gland	Carcinoma	1.5 T	Retrospective	Hospital	45 (Male) / 55 (Fem)	55.6	100	34.0%
Sumi et al. 2012 (DW MRI)[50]	Japan	Asia	11	31	20	Salivary glands	Not indicated	1.5 T	Retrospective	Hospital	16 (Male) / 15 (Fem)	61	31	35.4%
Sumi et al. 2014 (DW and DCE MRI)[49]	Japan	Asia	14	36	22	Not indicated	Lymphoma and Carcinoma	1.5 T	Retrospective	Hospital	Not indicated	Not indicated	Not indicated	38.8%
Takashima et al. 1997 (traditional MRI)[51]	Japan	Asia	14	53	39	Parotid gland	Carcinoma	1.5 T	Retrospective	Hospital	25 (Male) / 28 (Fem)	55.3	53	26.4%
Turner et al. 2008 (DW and traditional MRI)[52]	France	Europe	7	29	22	Parotid gland	Carcinoma and Adenocarcinoma	Not indicated	Retrospective	Not indicated o	17 (Male) / 12 (Fem)	47	29	24.1%
Yabuuchi et al. 2003 (DCE MRI)[53]	Japan	Asia	11	33	22	Salivary glands	Carcinoma and Adenocarcinoma	0.5 T	Retrospective	Not indicated	13 (Male) / 16 (Fem)	59	29	33.3%
Yerli et al. 2010 (traditional MRI)[54]	Turkey	Asia	5	25	20	Parotid gland	Carcinoma	1.5 T	Retrospective	Hospital	9 (Male) / 16 (Fem)	66.1	25	20.0%
**TOTAL**	**-**	**-**	**377**	**1,404**	**1,028**	**-**	**-**	**-**	**-**	**-**	**-**	**MEAN** **55.4**	**MEAN** **59.1**	**MEAN** **29.1%**

Relative to DW MRI, the global sensitivity was 76.4% (95% CI: 67.3%–83.9%), and the global specificity was 91.3% (95% CI: 87.3%–94.4%). The global positive likelihood ratio was 8.0 (95% CI: 3.7–17.4), i.e., a positive DW MRI result increased by 8.0-fold the odds of an accurate diagnosis of patients who actually had oral cancer. Heterogeneity was found both with the Cochran’s Q test (p = 0.0067) and using *τ*^2^ = 0.7296; inconsistency (*I*^2^ = 62.2%) was moderate [27]. The global negative likelihood ratio was 0.3 (95% CI: 0.20–0.50). This result indicates the use of DW MRI given that it is close to zero (i.e., the odds of a false-positive result is only increased by a factor of 0.3). Cochran’s Q test did not detect heterogeneity, but a *τ*^2^ of 0.1647 indicates heterogeneity; inconsistency (*I*^2^ = 39.9%) was moderate [27]. Global DOR was 30.7 (95% CI: 12.7–74.3), i.e., the odds of a positive DW MRI result were 30.7-fold higher among individuals with oral cancer compared to those without disease. The area under the SROC was high [55] (AUC = 0.917; 95% CI: 0.915–0.918), and Q* was 0.85 for DW MRI for the diagnosis of oral cancer.

Relative to DCE MRI, the global sensitivity was 84.0% (95% CI: 76.2%–90.1%), and the global specificity was 89.5% (95% CI: 84.7%–93.2%). The global positive likelihood ratio was 7.2 (95% CI: 3.1–16.6); therefore, a positive DCE MRI result increased by 7.2-fold the odds of an accurate diagnosis of patients who actually had oral cancer. Heterogeneity was found both on Cochran’s Q test (p = 0.0002) and the *τ*^2^ value (0.8916); inconsistency (*I*^2^ = 77.1%) was high [27]. The global negative likelihood ratio was 0.2 (95% CI: 0.1–0.3). This result indicates the use of DCE MRI given that this value is close to zero. Specifically, the odds of a false-positive result are increased by only a factor of 0.22. Heterogeneity was not detected in Cochran’s Q test (0.2588) or by the *τ*^2^ value (0.0951); inconsistency (*I*^2^ = 22.4%) was low [27]. Global DOR was 48.1 (95% CI: 22.4–103.2), i.e., the odds of a positive DCE MRI result were 48.1-fold higher among individuals with oral cancer compared to those without the disease. The area under the SROC was high[55] (AUC = 0.936; 95% CI: 0.934–0.937), and Q* was 0.87 for DCE MRI for the diagnosis of oral cancer.

Regarding traditional MRI, the global sensitivity was 72.5% (95% CI: 66.4%–78.0%), and the global specificity 86.6% (95% CI: 84.0%–88.9%). The global positive likelihood ratio was 6.5 (95% CI: 3.8–11.0), i.e., a positive traditional MRI result increased by 6.5-fold the odds of an accurate diagnosis of patients who actually had oral cancer. Heterogeneity was found on Cochran’s Q test (p<0.001) and was confirmed by the *τ*^2^ value (0.6480); and inconsistency (*I*^2^ = 83.1%) was high[27]. The global positive likelihood ratio was 0.3 (95% CI: 0.2–0.4), a result favouring the use of traditional MRI given that the value was close to zero. Specifically, the odds of a false-positive result are increased by only 0.3-fold. Heterogeneity was detected using Cochran’s Q test (p = 0.0005) and was confirmed by the *τ*^2^ value (0.2273); inconsistency (*I*^2^ = 65.3%) was moderate[27]. The global DOR was 23.9 (95% CI: 13.2–43.3); therefore, the odds of a positive traditional MRI result were 23.9-fold higher among individuals with oral cancer compared to those without disease. The area under the SROC was high[55] (AUC = 0.894; 95% CI: 0.894–0.895), and Q* was 0.82 for traditional MRI for the diagnosis of oral cancer.

**Fig 2:**
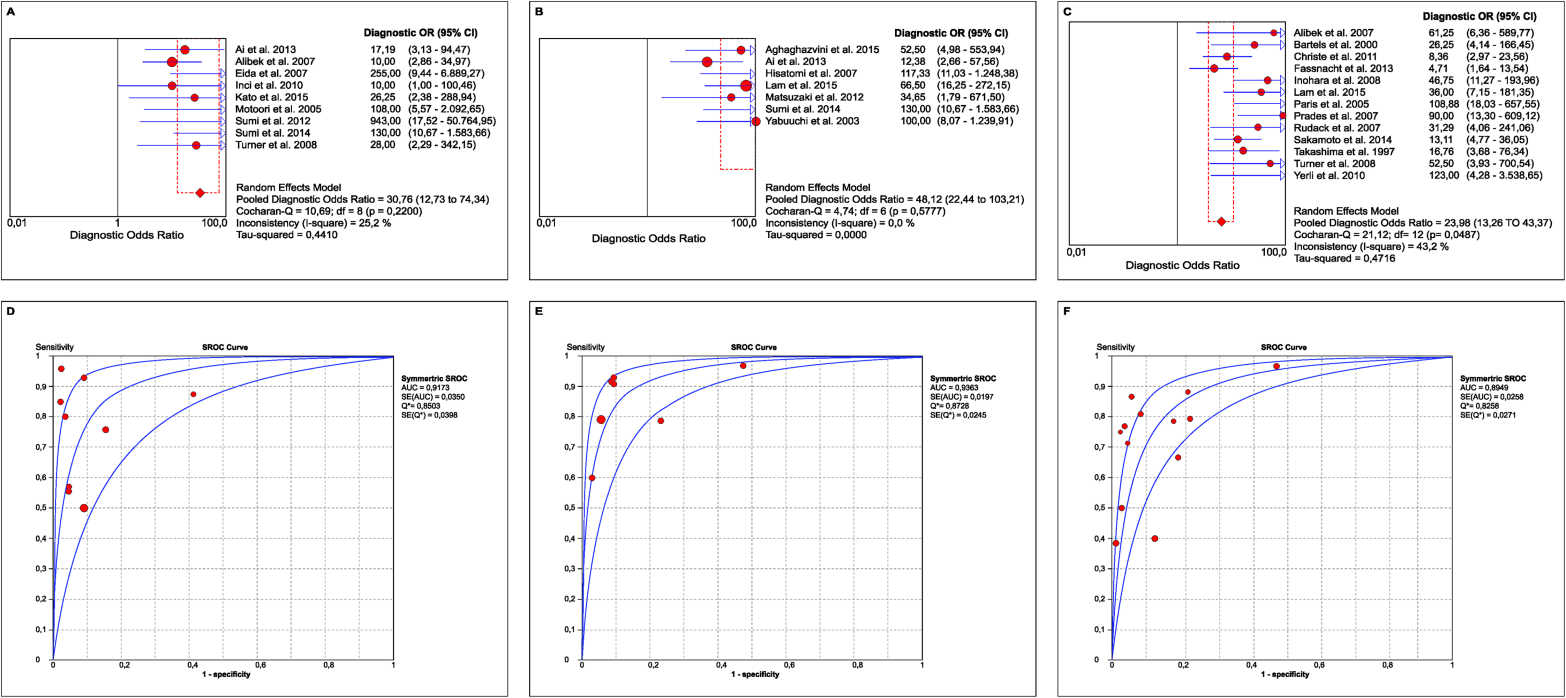
Measures of diagnostic performance.

The data in Table 2 indicate that DCE MRI has the highest sensitivity, DOR and AUC and that DW MRI has the highest specificity. In turn, traditional MRI exhibited the lowest values for all of the parameters considered, in addition to a possible threshold effect (not confirmed by the ROC panel–Fig 2).

**Table 2:** Summary of diagnostic measures.

MRI	Diagnostic measures				Diagnostic threshold				
	Sensitivity (95% CI)	Specificity (95% CI)	DOR (95% CI)	AUC (95% CI)	r_s_	P	B	P	ROC panel
DW MRI	76.4% (67.3%-83.9%)	91.3% (87.3%-94.4%)	30.7 (12.7–74.3)	0.917 (0.915–0.918)	-0.168	0.666	0.018	0.9569	No diagnostic threshold effect
DCE MRI	84.0% (76.2%-90.1%)	89.5% (84.7%-93.2%)	48.1 (22.4–103.2)	0.936 (0.934–0.937)	0.577	0.175	-0.086	0.7936	No diagnostic threshold effect
Traditional MRI	72.5% (66.4%-78.0%)	86.6% (84.0%-88.9%)	23.9 (13.2–43.3)	0.894 (0.894–0.895)	0.610	0.027*	-0.085	0.6373	No diagnostic threshold effect

*statistically significant (p<0.05).

The data in Table 2 suggest a threshold effect with traditional MRI, as shown by a positive correlation between the logit of sensitivity and [1 –specificity] (p = 0.027). However, visual inspection of the ROC panel does not indicate a threshold effect in any of the three types of MRI analysed (**Fig 2**).

According to meta-regression analysis (Table 3), the magnetic field strength might influence the accuracy (relative DOR [RDOR] = 0.32; 95% CI: 0.13–0.82; p = 0.0233) of traditional MRI. This finding may account for the heterogeneity exhibited by this MRI type. Regarding the other two MRI types, the results described in Table 3 do not indicate that the analysed cofactors are associated with heterogeneity.

**Table 3:** Sensitivity analysis.

Sensitivity analysis				Meta-regression				
Covariable	N (studies)	DOR (95% CI)	*I*^*2*^	Covariable	Coefficient	Standard error	RDOR (95% CI)	P
**DW MRI**				**DW MRI**				
**Continent**				Age	0.060	0.0462	1.06 (0.95–1.19)	0.2423
Europe	3	11.81 (4.31–32.35)	0%	Prevalence	0.003	0.0232	1.00 (0.95–1.06)	0.9106
Asia	6	59.77 (19.72–181.11)	10.6%	Sample size (n)	-0.018	0.0100	0.98 (0.96–1.01)	0.1152
**Tumour site**				Magnetic field strength	-0.222	1.1056	0.80 (0.05–11.98)	0.8476
Parotid gland	5	16.06 (6.62–38.95)	0%					
Other	4	89.43 (15.83-505-17)	40.1%					
**DW global**	9	30.76 (12.73–74.34)	25.2%					
**DCE MRI**				**DCE MRI**				
**Tumour site**				Age	0.087	0.0454	1.09 (0.94–1.26)	0.1505
Salivary glands	4	75.04 (27.89–201.96)	0%	Prevalence	-0.037	0.0200	0.96 (0.92–1.01)	0.1213
Other	3	29.99 (6.98–128.80)	23.7%	Sample size (n)	0.003	0.0164	1.00 (0.96–1.05)	0.8449
**DCE global**	9	48.12 (22.44–103.21)	0%	Magnetic field strength	-0.018	0.0729	0.98 (0.91–1.18)	0.8165
**TRADITIONAL MRI**				**TRADITIONAL MRI**				
**Continent**				Age	-0.205	0.1052	0.81 (0.64–1.04)	0.0868
Asia	4	21.40 (11.05–41.44)	0%	Prevalence	-0.022	0.0356	0.98 (0.91–1.06)	0.5569
Americas	2	11.36 (4.20–30.70)	11%	Sample size (n)	-0.016	0.0094	0.98 (0.96–1.00)	0.1191
Europe	7	39.45 (11.79–132.03)	61.7%	Magnetic field strength	-1.129	0.4141	0.32 (0.13–0.82)	0.0233*
**Tumour site**								
Parotid gland	11	23.48 (11.91–46.29)	50.4%					
Salivary glands	2	34.11 (9.60-121-10)	0%					
**Traditional global**	13	23.98 (13.26–43.37)	43.2%					

Sensitivity analysis revealed a slight reduction in inconsistency in some subgroups (Table 3). For DW MRI, the heterogeneity of studies conducted in Europe and for tumours located in the parotid gland decreased (*I*^*2*^ fell from 25.2% to 0%). As concerns DCE MRI, heterogeneity was maintained in the cases of salivary glands tumours and increased for tumours in other sites. Lastly, in regard to traditional MRI, heterogeneity decreased in the studies conducted in Asia (*I*^*2*^ fell from 43.2% to 0%) and the Americas (*I*^*2*^ decreased from 43.2% to 0%) and when the tumour was located in the salivary glands (*I*^*2*^ decreased from 43.2% to 0%).

The methodological quality of the studies was very high, although some QUADAS-2 items scored negatively or the corresponding data were not presented by the authors.

The present is the first systematic review that assessed the accuracy of MRI for the diagnosis of oral cancer. The results show that the accuracy of the index test was good according to DOR (DW MRI– 30.7; DCE MRI– 48.1; traditional MRI– 23.9) and SROC-AUC (DW MRI– 0.91; DCE MRI– 0.93; traditional MRI– 0.89) analyses. In addition, MRI is non-invasive and can therefore be used as an auxiliary means for diagnosis and prevention of oral cancer, as well as for the planning of surgical interventions.

The 24 studies analysed in the present systematic review indicate the ability of MRI to diagnose malignant tumours with adequate sensitivity and specificity in patients with suspected oral cancer.

Our study extracted data from retrospective diagnostic accuracy studies. The methodological quality of the studies was very high, although some QUADAS-2 [20, 21] items scored negatively or the corresponding data were not presented by the authors **(S1 Table)**. We performed bivariate analysis of sensitivity, specificity, likelihood ratio and DOR (with 95% CI); the latter considers intra- and inter-study variability. We applied the latest Cochrane guidelines for systematic reviews and diagnostic test accuracy studies [21–23, 25, 28].

MRI is the technique that provides the highest-quality images of soft tissues, without employing ionising radiation and without known biological risks [12, 56]. MRI also enables multiplanar studies (axial, coronal and sagittal) and provides images with high anatomical definition. The very low section thickness allows visualising structural lesions on the order of millimetres. Currently available clinical MRI devices use magnetic fields of 0.2 T to 3.0 T, and magnetic field strength is directly proportional to the definition of the obtained images and to the applicability of additional applications (e.g., software) [6].

Some researchers observed that the application of imaging tests to head and neck malignant neoplasms has substantially increased in recent years with the development of the modern, last-generation methods [11]. Diagnostic imaging techniques, such as computed tomography, ultrasound, MRI, bone scintigraphy and positron emission tomography (PET), must be used to complement the data obtained on clinical examination in terms of the tumour primary site, extension and invasion of adjacent structures [12].

Recently, DW MRI was suggested to be a highly sensitive method for assessment of the response to treatment, as well as for monitoring head and neck cancer [57]. Subsequently, several studies applied DW MRI as a diagnostic test for several brain conditions [58, 59]. In addition, this technique was applied in studies of the spinal cord and vertebral column [60], liver [61], kidneys and urinary tract [62].

Our study detected high heterogeneity among DW MRI results. A possible cause for such heterogeneity is variation in the magnetic field strength applied, which was 1.5 T in seven studies, 3 T in one study and unspecified in another. The reason for such heterogeneity that the magnetic field strength is directly related to the quality and wealth of detail of the obtained images. Padhani et al. [63] reported that accepted standards in DW MRI measurement or analysis methods have not been established. Moreover, current imaging analysis protocols suggested by different companies vary. This is the case even when considering the same manufacturer, which can alter its suggested methods following upgrades [61].

DCE MRI is a promising imaging technique for the assessment of microvascular parameters of tissue perfusion and consequently hypoxia [64]. This method is a non-invasive radiological technique that provides information on the microvascular environment of cancer lesions through the analysis of the contrast medium kinetics [65]. This test has potential for use in the detection and characterisation of tumours [66], as well as in the planning of treatment, the initial prediction of the response to treatment and assessment of treatment results [65]. We also found heterogeneity among results for this imaging modality, possibly due to variation in the cancer lesion sites assessed with DCE MRI. Specifically, the analysed region was the salivary glands in four studies, the palate in one and the tongue in another; the location was unspecified in one study.

In turn, traditional MRI has an outstanding role in the establishment of the origin, location and boundaries of lesions. Conventional radiographs have limitations, only being useful for screening lesions adjacent to mineralised tissue. This type of MRI does not employ ionising radiation and is one of the most indicated tests for the diagnosis of tumour lesions [67–73]. Considerable heterogeneity was also found among traditional MRI studies in the present meta-analysis, which might be accounted for by the various types of cancer assessed, different lesion sites and differences in calibration.

## Conclusions

Providing integral care to users of public and private health services is a highly relevant goal, as are diagnostic confirmation in cases of oral cancer, pre-treatment assistance and control of the progression of disease once it is diagnosed. For these reasons, the present systematic review assessed the accuracy of MRI for the diagnosis of oral cancer, concluding this method is adequate for the intended purpose. The three types of MRI assessed (DW, DCE and traditional MRI) exhibited satisfactory accuracy compared to biopsy.

MRI is an adequate diagnostic option because it is available at public health services (in the case of Brazil); it can be used for monitoring patients as well as for obtaining a precise delimitation of lesions.

However, the variability among the currently available MRI methods has hindered their selection for clinical use; this bias was evident in the analysed studies given that the type of MRI used was not standardised.

Heterogeneity was found among the analysed studies, likely due to variation of the magnetic field strength used for traditional MRI as well as of the criteria for malignancy.

Early treatment and screening is important for oral cancer patients, and better health care results in improved survival rates, a better quality of life and the possibility of reducing the number of unnecessary biopsies. For these reasons and based on the results of the present study, we suggest the pilot use of MRI, as an auxiliary and complementary method to biopsy for pre-treatment and monitoring of oral cancer patients.

## Supporting information

S1 Fig Full electronic search strategy–Pubmed. (DOCX)

S1 File Prisma checklist. (DOC)

S1 Table Results of the risk of bias assessment for each study, according to Quality Assessment of Diagnostic Accuracy Studies (QUADAS). (DOCX)
