# Supplementary material for: Meta-analysis of magnetic resonance imaging accuracy for diagnosis of oral cancer
Source: PLoS One. 2017 May 24;12(5):e0177462. doi: 10.1371/journal.pone.0177462 (PMC5443513; doi:10.1371/journal.pone.0177462)
Supplement: S1 Fig — (DOCX) [file pone.0177462.s001.docx]

**S1 Fig. Full electronic search strategy - Pubmed**

1. sensitiv* OR specifici* OR "Sensitivity and Specificity"[Mesh] OR(predictive AND value*) OR "Predictive Value of Tests"[Mesh] OR accuracy* OR “False Negative*” OR “False Positive*”
2. "Mouth Neoplasms"[Mesh] OR “Mouth Neoplasm” OR “Neoplasm, Mouth” OR “Neoplasms, Oral” OR “Neoplasm, Oral” OR “Oral Neoplasm” OR “Oral Neoplasms” OR “Neoplasms, Mouth” OR “Cancer of Mouth” OR “Mouth Cancers” OR “Mouth Cancer” OR “Cancer, Mouth” OR “Cancers, Mouth” OR “Oral Cancer” OR “Cancer, Oral” OR “Cancers, Oral” OR “Oral Cancers” OR “Cancer of the Mouth” OR “Buccal Cancer”)
3. "Magnetic Resonance Imaging" [Mesh] OR "Magnetic Resonance Imaging" OR “Imaging, Magnetic Resonance” OR “NMR Imaging” OR “Imaging, NMR” OR Zeugmatography OR “Tomography, MR” OR “Tomography, NMR” OR “MR Tomography” OR “NMR Tomography” “Tomography, Proton Spin” OR “Proton Spin Tomography” OR “Magnetization Transfer Contrast Imaging” OR “MRI Scans” OR “MRI Scan” OR “Scan, MRI” OR “Scans, MRI” OR fMRI OR “MRI, Functional” OR “Functional MRI” OR “Functional MRIs” OR “MRIs, Functional” OR “Functional Magnetic Resonance Imaging” OR “Magnetic Resonance Imaging, Functional” OR “Imaging, Chemical Shift” OR “Chemical Shift Imagings” OR “Imagings, Chemical Shift” OR “Shift Imaging, Chemical” OR “Shift Imagings, Chemical” OR “Chemical Shift Imaging”

4. #1 AND #2 AND #3
