# Supplementary material for: Meta-analysis of magnetic resonance imaging accuracy for diagnosis of oral cancer
Source: PLoS One. 2017 May 24;12(5):e0177462. doi: 10.1371/journal.pone.0177462 (PMC5443513; doi:10.1371/journal.pone.0177462)
Supplement: S1 Table — (DOCX) [file pone.0177462.s003.docx]

**S1 Table. Results of the risk of bias assessment for each study, according to Quality Assessment of Diagnostic Accuracy Studies (QUADAS).**

| **Study** | **Risk of Bias Applicability** | | | | | | |
| --- | --- | --- | --- | --- | --- | --- | --- |
|  | **Patient Selection** | **Index Test** | **Reference Standard** | **Flow and Timing** | **Patient Selection** | **Index Test** | **Reference Standard** |
| Aghaghazviniet al. 2015 (DCE MRI)[31] | ? | ☺ | ☺ | ☺ | ☺ | ☺ | ☺ |
| Ai et al. 2013 (DCE and DW MRI)[32] | ☺ | ☺ | ☺ | ☺ | ☺ | ☺ | ☺ |
| Alibek et al. 2007 (DW and traditional MRI)[33] | ? | ☺ | ☺ | ☺ | ? | ☺ | ☺ |
| Bartels et al. 2000 (traditional MRI)[34] | ? | ☺ | ☺ | ☺ | ☺ | ☺ | ☺ |
| Christe et al. 2011 (traditional MRI)[35] | ☺ | ☺ | ☺ | ☺ | ☺ | ☺ | ☺ |
| Eida et al. 2007 (DW MRI)[36] | ☺ | ☺ | ☺ | ? | ☺ | ☺ | ☺ |
| Fassnacht et al. 2013 (traditional MRI)[38] | ☺ | ☺ | ☺ | ☺ | ? | ☺ | ☺ |
| Hisatomi et al. 2007 (DCE MRI)[39] | ☺ | ☺ | ☺ | ? | ☺ | ☺ | ☺ |
| Inci et al. 2010 (DW MRI)[37] | ☺ | ☹ | ☺ | ☺ | ☺ | ☺ | ☺ |
| Inohara et al. 2008 (traditional MRI)[40] | ☺ | ☺ | ☺ | ☺ | ☺ | ☺ | ☺ |
| Kato et al. 2015 (DW MRI)[41] | ☺ | ☺ | ☺ | ? | ☺ | ☺ | ☺ |
| Lam et al. 2015 (DCE and traditional MRI)[42] | ☺ | ☺ | ☺ | ☺ | ☺ | ☺ | ☺ |
| Matsuzaki et al. 2012 (DCE MRI)[43] | ☺ | ☺ | ☺ | ? | ☺ | ☺ | ☺ |
| Motoori et al. 2005 (DW MRI)[44] | ☺ | ☺ | ☺ | ☺ | ☺ | ☺ | ☺ |
| Paris et al. 2005 (traditional MRI)[45] | ☺ | ☺ | ☺ | ☺ | ☺ | ☺ | ☺ |
| Prades et al. 2007 (traditional MRI)[46] | ☺ | ☺ | ☺ | ☺ | ☺ | ☺ | ☺ |
| Rudack et al. 2007 (traditional MRI)[47] | ? | ☺ | ☺ | ☺ | ☺ | ☺ | ☺ |
| Sakamoto et al. 2014 (traditional MRI)[48] | ☺ | ☺ | ☺ | ☺ | ☺ | ☺ | ☺ |
| Sumi et al. 2012 (DW MRI)[50] | ☺ | ☺ | ☺ | ☺ | ☺ | ☺ | ☺ |
| Sumi et al. 2014 (DW and DCE MRI)[49] | ☺ | ☺ | ☺ | ☺ | ☺ | ☺ | ☺ |
| Takashima et al. 1997 (traditional MRI)[51] | ☹ | ☺ | ☺ | ☺ | ☺ | ☺ | ☺ |
| Turner et al. 2008 (DW and traditional MRI)[52] | ☺ | ☺ | ☺ | ? | ☺ | ☺ | ☹ |
| Yabuuchi et al. 2003 (DCE MRI)[53] | ☹ | ☺ | ☺ | ☺ | ☺ | ☺ | ☺ |
| Yerli et al. 2010 (traditional MRI)[54] | ☹ | ☺ | ☺ | ☺ | ☺ | ☺ | ☺ |
| ☺Low risk ☹High risk ? Unclear | | | | | | | |
